# Outcomes of early oral antibiotic transition for the treatment of uncomplicated enterococcal bacteremia

**DOI:** 10.1017/ash.2026.10746

**Published:** 2026-06-15

**Authors:** Cecilia Li, Madeline Schultze, Carissa Tedeschi, Alex Rock, Eric Gillett, Alyssa Letourneau, Tiffany Wu

**Affiliations:** 1 Department of Pharmacy, Massachusetts General Hospital, Boston, USA; 2 Department of Pharmacy, Brigham and Women’s Hospital, Boston, USA; 3 Department of Medicine, Massachusetts General Hospital, Boston, USA

## Abstract

**Objective:**

To compare clinical outcomes in adults with uncomplicated enterococcal bacteremia treated with intravenous (IV) therapy alone versus an early oral (PO) transition and evaluate treatment characteristics and safety outcomes.

**Design:**

Retrospective cohort study between January 1, 2021, and August 1, 2024. The primary outcome was clinical cure at 90 days after antibiotic completion. Secondary outcomes included treatment failure, infection-related readmission, hospital length of stay (HLOS), and 90-day mortality.

**Setting:**

Massachusetts General Hospital and Brigham and Women’s Hospital, two tertiary care centers in Boston, MA.

**Patients:**

Adult patients with at least one positive blood culture with *Enterococcus* spp. Patients were excluded if they had polymicrobial or persistent bacteremia, invasive infection, incomplete source control, died before completing therapy, or received fewer than 7 days of antibiotics.

**Results:**

209 patients were included, of which 119 received IV therapy alone and 90 underwent early PO transition. Gastrointestinal (41.6%) and genitourinary (32.1%) sources were most common, and 86 patients (41.1%) had immunocompromising conditions. Clinical cure was similar between the IV and early PO transition groups (73.9% vs 82.2%; *P* = .156), with no significant differences in secondary and safety outcomes. HLOS was significantly shorter in the early PO transition group (9 days vs 20 days; *P* < .001).

**Conclusion:**

Early PO transition appears to be a safe and effective strategy for uncomplicated enterococcal bacteremia and was associated with reduced HLOS.

## Background

Enterococcal bacteremia is a significant finding that warrants prompt evaluation for an infection source and metastatic sites of disease. Although severe complications such as infective endocarditis can occur, many episodes present without metastatic findings when identified early, often arising from gastrointestinal (GI) or genitourinary (GU) tracts or from intravascular lines.^1^ Current practice for these less severe infections typically favors 7–14 days of intravenous (IV) antibiotic therapy, consistent with the 2009 IDSA Clinical Practice Guideline for the Diagnosis and Management of Intravascular Catheter-Related Infections, which recommends 7–14 days of treatment for uncomplicated catheter-related enterococcal bloodstream infections with adequate source control.^2^ Despite the frequency of these less complicated presentations, no standardized definition or management framework for uncomplicated enterococcal bacteremia has been established. These uncertainties have generated interest in shorter total treatment courses and earlier transitions to highly bioavailable oral (PO) antibiotics to complete therapy.^3–5^

Evidence from other uncomplicated gram-positive bacteremia studies, such as those caused by *Streptococcus* spp. and select cases of *Staphylococcus aureus*, demonstrates that early PO transition can be a safe and effective treatment strategy while reducing hospital length of stay and IV line-associated complications.^6–10^ Early PO transition is defined as the receipt of initial IV antibiotic therapy followed by transition to PO antibiotic therapy to complete the planned treatment course. Findings from prior studies highlight the need to determine whether similar approaches can be safely applied to uncomplicated enterococcal bacteremia. This study compares clinical outcomes in adults with uncomplicated enterococcal bacteremia treated with IV therapy alone versus an early PO transition.

## Methods

### Study subjects

We conducted a multicenter, retrospective cohort study of adult patients who had at least one blood culture positive with an *Enterococcus* spp. between January 1, 2021, and August 1, 2024, at Massachusetts General Hospital (MGH) and Brigham and Women’s Hospital (BWH), two large tertiary medical centers in Boston, Massachusetts. All *Enterococcus* isolates from blood were included regardless of species or susceptibility pattern. The study protocol was approved by the Institutional Review Board (IRB) at both institutions with a waiver of informed consent. Patients were included if they were ≥18 years old and had clearance of blood cultures within 48 hours after initiation of effective antibiotics. Eligible sources included GU, GI, skin and soft tissue, and line-associated infections with established source control within 72 hours of index blood culture, if applicable. Sources of infection and necessary diagnostic workup were determined by clinician documentation, with infectious diseases consult notes used preferentially when available. Cases with an unknown source were included if the treating physician documented the absence of metastatic infection with no concerns for an uncontrolled source in the electronic medical record (EMR).

Complicated enterococcal bacteremia cases were excluded and defined as polymicrobial bacteremia, including two distinct *Enterococcus* isolates in the same culture; persistent bacteremia defined as positive blood cultures for ≥48 hours after initiation of effective antibiotics; invasive enterococcal infection such as endocarditis or osteomyelitis; or had incomplete source control. Patients were also excluded if they transitioned to comfort measures only or died prior to therapy completion, received fewer than 7 total days of antibiotic therapy, were unable to take oral medications, or lacked a suitable oral option based on drug susceptibility, allergy, or intolerance.

### Data collection

Patients were identified from positive blood culture reports generated through SAP Crystal Reports from the Microbiology Laboratory Information System (Sunquest). Demographics, treatment characteristics, microbiology, and outcomes were collected by retrospective EMR review and stored in REDCap.^11^

### Outcomes and definitions

The primary outcome was clinical cure 90 days after completion of antibiotic therapy, defined as survival without infection-related readmission or treatment failure. Treatment failure was defined as provider-determined clinical non-improvement, infection recurrence as evidenced by isolation of the same organism on clinical culture, and/or clinical worsening prompting a change in therapy. Secondary outcomes included individual components of the primary outcome, hospital length of stay (HLOS), and total treatment duration, measured from the first day of effective antibiotics to the last day of planned therapy. Safety outcomes, including antibiotic-associated adverse events and IV line-associated complications, were assessed. Adverse events were identified using predefined EMR search terms, such as “*Clostridioides difficile*” and “nausea.” Serum creatinine (SCr) and creatine kinase trends were reviewed for acute kidney injury and myopathy, respectively.

Potential risk factors for adverse outcomes were collected, including injection drug use, Charlson Comorbidity Index (CCI), Pitt bacteremia score (PBS), and immunocompromising conditions.^12^ Patients were classified as having an immunocompromising condition if they met any of the following criteria: solid tumor malignancy with chemotherapy in the past 90 days, solid organ transplant (SOT) recipient with ongoing immunosuppression therapy, hematopoietic stem cell transplant (HSCT) recipient, neutropenia (absolute neutrophil count ≤ 500 cells/µL) at time of blood culture positivity, person living with human immunodeficiency virus (PLWH) with a CD4 count ≤ 200 cells/µL, or receiving high-dose corticosteroids, defined as a prednisone equivalent dose of ≥20 mg/day for at least 14 days. Mild renal disease was defined as a SCr level between 2–3 mg/dL; moderate renal disease was defined as a SCr > 3 mg/dL; and severe renal disease was defined as dialysis dependent, prior renal transplant, or presence of uremia.

### Data analysis and statistical methods

Descriptive statistics were used to summarize patient demographics and infection characteristics. Categorical variables were reported as counts and percentages. Continuous variables were reported as means with standard deviations or as medians with interquartile ranges (IQR, 25^th^ – 75^th^ percentile) depending on normality. Categorical variables were analyzed using chi-squared test or Fisher’s exact test where appropriate. The Mann-Whitney *U* test was used to compare nonparametric, continuous variables. A univariate logistic regression was performed to evaluate associations between baseline characteristics and the likelihood of early PO transition. Variables with a *P* value of <.2 in the univariate analysis, along with clinically important covariates, were included in a multivariable logistic regression model. The GU tract was selected a priori as the reference category for source of infection given its frequency as a source of enterococcal bacteremia. Odds ratios (OR) with 95% confidence intervals (CI) were reported for all variables in the final model, and model calibration was assessed using the Hosmer-Lemeshow goodness-of-fit test. Statistical analyses were conducted using SPSS Statistics for Windows, version 31.0.^13^

## Results

### Baseline characteristics

**Figure 1.**
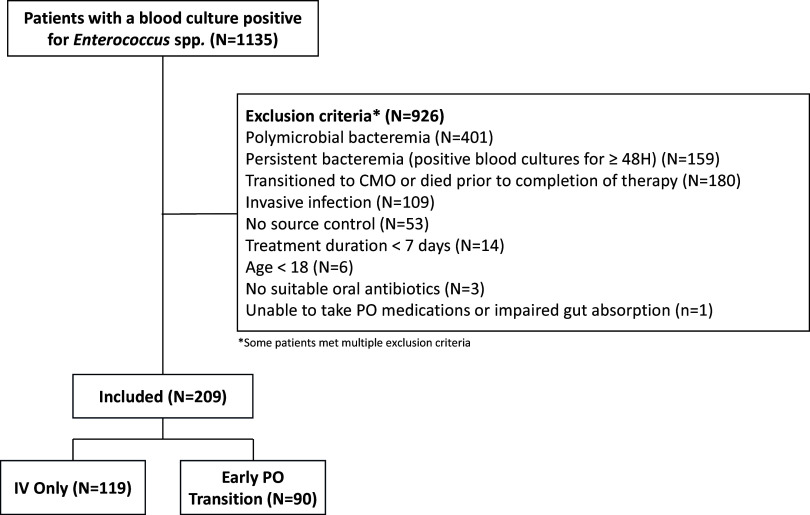
Patient selection and exclusion criteria.

During the study period, 1,135 patients with enterococcal bacteremia were reviewed, and 209 patients (18.4%) were included in the final analysis. Reasons for exclusion are outlined in Figure 1. Among included patients, 119 (56.9%) received IV antibiotics alone and 90 (43.1%) received an early PO transition. Patients with GU sources were more likely to receive an early PO transition, whereas patients with line-related sources were more likely to receive IV therapy. Overall, 86 patients (41.1%) had an immunocompromising condition, most commonly solid tumor malignancy treated with chemotherapy within 90 days before index culture. 25 patients (12%) had an ANC < 500 cells/uL at the time of index blood culture positivity. The most common pathogen was *E. faecalis* (61.2%), followed by *E. faecium* (38.8%). No other *Enterococcus* species were identified. Patient and treatment characteristics are further detailed in Table 1.

**Table 1.** Patient and treatment characteristics

Characteristic	Overall (n = 209)	IV only (n = 119)	Early PO transition (n = 90)	*P*-value
Age (years), median (IQR)	68 (56.5–75)	68 (56–75)	67 (57–74)	.518
Male, n (%)	120 (57.4)	70 (58.8)	50 (55.6)	.636
White, n (%)	161 (77.0)	92 (77.3)	69 (76.7)	.913
BMI (kg/m^2^), median (IQR)	26.5 (23.1–31.6)	25.9 (22.9–31.2)	27.6 (23.4–31.7)	.316
Charlson comorbidity index, median (IQR)	5 (4–8)	6 (4–7)	5 (4–8)	.841
Diabetes, n (%)	59 (28.2)	32 (26.9)	27 (30.0)	.621
Renal Function, n (%)				
Normal	162 (77.5)	89 (74.8)	73 (81.1)	.278
Mild CKD	16 (7.7)	8 (6.7)	8 (8.9)	.560
Moderate CKD	10 (4.8)	8 (6.7)	2 (2.2)	.193
Severe CKD	21 (10.0)	14 (11.8)	7 (7.8)	.342
Immunocompromising condition, n (%)	86 (41.1)	51 (42.9)	35 (38.9)	.564
Solid tumor malignancy with cytotoxic chemotherapy in the past 90 days	32 (15.3)	20 (16.8)	12 (13.3)	–
SOT recipient on immunosuppression	21 (10.0)	11 (9.2)	10 (11.1)	–
Hematologic malignancy	23 (11.0)	12 (10.1)	11 (12.2)	–
HSCT recipient	6 (2.9)	6 (5.0)	0 (0)	–
High dose steroids	4 (1.9)	2 (1.7)	2 (2.2)	–
ANC < 500 cells/uL	25 (12.0)	15 (12.6)	10 (11.1)	.742
Pitt Bacteremia Score, median (IQR)	1 (0–2)	1 (0–2)	1 (0–2)	.219
PWID, n (%)	0 (0)	0 (0)	0 (0)	–
Source of infection, n (%)				
Gastrointestinal	87 (41.6)	52 (43.7)	35 (38.9)	.485
Genitourinary	67 (32.1)	27 (22.7)	40 (44.4)	<.001
Line-related infection	36 (17.2)	28 (23.5)	8 (8.9)	.006
Skin and soft tissue	2 (1.0)	1 (0.8)	1 (1.1)	1.000
Unclear^†^	17 (8.1)	11 (9.2)	6 (6.7)	.500
Pathogen, n (%)				
Amp-S *E. faecalis*	128 (61.2)	76 (63.9)	52 (57.8)	.371
Amp-R *E. faecalis*	0 (0)	0 (0)	0 (0)	–
Amp-S *E. faecium*	19 (9.1)	11 (9.2)	8 (8.9)	.930
Amp-R *E. faecium*	62 (29.7)	31 (26.1)	31 (34.4)	.188
Infectious diseases consult, n (%)	176 (84.2)	106 (89.1)	70 (77.8)	.027
Total antibiotic duration (days), median (IQR)	14 (13–16)	15 (12–16)	14 (13–16)	.966
IV antibiotic duration (days), median (IQR)	11 (5.5–15)	15 (12–16)	5 (3–8)	<.001
Time to oral therapy initiation (days), median (IQR)	5 (3–9)	N/A	5 (3–9)	–
PO antibiotic duration (days), median (IQR)	9 (6–12)	N/A	9 (6–12)	–

BMI, body mass index; CKD, chronic kidney disease; SOT, solid organ transplant; HSCT, hematopoietic stem cell transplant; ANC, absolute neutrophil count; PWID, persons who inject drugs.

† Deemed uncomplicated by treating provider (e.g., no invasive infection or uncontrolled source); Amp-S, ampicillin-susceptibile; Amp-R, ampicillin-resistant.

### Treatment characteristics

Among the 209 patients, more patients in the IV group received infectious diseases (ID) consultation compared to the early PO transition group (89.1% vs 77.8%; *P* = .027). The median total antibiotic durations were similar between the IV only and early PO transition groups (15 d [IQR 12–16] vs 14 d [IQR 13–16]; *P* = .966). Among patients transitioned to PO therapy, the median time to PO initiation after starting IV antibiotics was 5 days (IQR 3–9), and the median duration of PO therapy was 9 days (IQR 6–12). Notably, 7 patients (8%) in the early PO transition group initiated oral antibiotics on day 1 of effective treatment. The most frequently prescribed oral antibiotic in the early transition group was linezolid (65.6%, 59/90), followed by amoxicillin (21.1%, 19/90). All oral antibiotic regimens used are listed in Table 2.

**Table 2.** Oral antibiotic regimens

Antibiotic	Dose	Frequency, n (%) (n = 90)
Linezolid	600 mg q12h	59 (65.6)
Amoxicillin	Total	19 (21.1)
	1,000 mg q8h	16 (17.8)
	1,000 mg q12h	1 (1.1)
	875 mg q12h	2 (2.2)
Amoxicillin/clavulanate	Total	6 (6.7)
	875/125 mg q12h	5 (5.6)
	250/125 mg q8h	1 (1.1)
Amoxicillin + Amoxicillin/clavulanate	1,000 mg q12h + 875/125 mg q12h	1 (1.1)
Doxycycline	100 mg q12h	1 (1.1)
Levofloxacin	750 mg q24h	2 (2.2)
	500 mg q24h	1 (1.1)
Tedizolid	200 mg q24h	1 (1.1)

If an antibiotic was renally dose-adjusted at the time of therapy initiation, doses were retrospectively adjusted at time of review to the equivalent doses for normal renal function.

### Outcomes

Clinical and safety endpoints are outlined in Table 3. Overall clinical cure was 77.5% and did not differ significantly between the IV and early PO transition groups (73.9% vs 82.2%, *P* = .156). Treatment failure occurred in 6.2% of patients, including 4.2% of patients in the IV only group and 8.9% in the early PO transition group (*P* = .165). There were 13 infection-related readmissions, with similar rates observed between groups (5.9% vs 6.7%, *P* = .816). All-cause mortality at 90 days was 15.8%, with no significant difference between the IV and the early PO transition group (18.5% vs 12.2%, *P* = .219). The median HLOS was significantly shorter in the early PO transition group compared with those who received IV therapy alone (9 d [IQR 6–15] vs 20 d [IQR 15–35], *P* < .001). Antibiotic-associated adverse effects were documented in 18 patients (8.6%) with no significant difference between the groups.

**Table 3.** Clinical and safety outcomes

Outcome	Overall (n = 209)	IV only (n = 119)	Early PO transition (n = 90)	*P*-value
Clinical cure, n (%)	162 (77.5)	88 (73.9)	74 (82.2)	.156
Treatment failure, n (%)	13 (6.2)	5 (4.2)	8 (8.9)	.165
Infection-related readmission, n (%)	13 (6.2)	7 (5.9)	6 (6.7)	.816
All-cause mortality at 90 days, n (%)	33 (15.8)	22 (18.5)	11 (12.2)	.219
Length of stay (days), median (IQR)	9 (6-15)	20 (15–35)	9 (6–15)	<.001
Antibiotic-associated adverse effects, n (%)	18 (8.6)	11 (9.2)	7 (7.8)	.541
* Clostridioides difficile* infection	3 (1.4)	3 (2.5)	0 (0)	–
Hypersensitivity reaction	4 (1.9)	2 (1.7)	2 (2.2)	–
Gastrointestinal effects	3 (1.4)	2 (1.7)	1 (1.1)	–
Acute kidney injury	4 (1.9)	3 (2.5)	1 (1.1)	–
Bone marrow suppression	3 (1.4)	1 (0.8)	2 (2.2)	–
IV-line complications	0 (0)	0 (0)	0 (0)	–
Myopathy	0 (0)	0 (0)	0 (0)	–
Other	1 (0.5)	0 (0)	1 (1.1)	–

### Logistic regression

In our univariable analysis, variables meeting the prespecified screening threshold (*P* < .20) included Pitt bacteremia score, ID consultation, and infectious source (GI, line-related, and unclear vs GU), as well as moderate renal dysfunction (vs none). Each of these variables was associated with lower odds of receiving early PO antibiotics. We also chose to include age, presence of an immunocompromising condition, and pathogen in the final multivariable regression model as clinically relevant patient and microbiologic-specific factors. In the final adjusted model (see Table 4 for the covariates included in the multivariable model), a higher Pitt bacteremia score (aOR 0.77 per point, 95% CI 0.62–0.97; *P* = .023), ID consultation (aOR 0.34, 95% CI 0.15–0.80; *P* = .014), GI versus GU source (aOR 0.29, 95% CI 0.14–0.62; *P* = .001), and line-related versus GU source (aOR 0.12, 95% CI 0.04–0.32; *P* < .001) were independently associated with lower odds of an early PO transition. *E. faecium* bacteremia (vs *E. faecalis*) was associated with higher odds of receiving early PO antibiotics (aOR 2.49, 95% CI 1.25–4.93; *P* = .009). Goodness-of-fit testing demonstrated adequate model performance (Hosmer-Lemeshow χ^2^ = 3.999, *P* = .857). Given the potential clinical correlation between severity of illness and likelihood of infectious disease consultation, we specifically assessed multicollinearity between the Pitt bacteremia score and ID consultation. Variance inflation factors (VIF) were 1.045 and 1.039 respectively, with tolerance values >0.95, indicating negligible collinearity. VIF and tolerance findings were consistent across all included variables.

**Table 4.** Univariate and multivariable regression model results for predictors of early PO transition Table 4 long description.

	Univariate model		Multivariate model	
Variable	OR with 95% CI	*P*-value	aOR with 95% CI	*P*-value
Male sex	1.14 (0.66–1.99)	.636	–	–
Age (per year)	0.99 (0.98–1.01)	.602	0.98 (0.96–1.01)	.143
BMI (per unit)	0.99 (0.97–1.03)	.935	–	**–**
Pitt bacteremia score (per point)	0.84 (0.69–1.02)	.085	0.77 (0.62–0.97)	.023
Immunocompromising condition	0.85 (0.49–1.48)	.564	0.85 (0.45–1.58)	.600
Renal dysfunction				
Mild vs. none	1.22 (0.44–3.41)	.705	–	–
Moderate vs. none	0.31 (0.06–1.48)	.141	–	–
Severe vs. none	0.61 (0.23–1.59)	.312	–	–
ID consultation	0.43 (0.20–0.92)	.029	0.34 (0.15–0.80)	.014
Pathogen (*E. faecium* vs. *E. faecalis*)	1.29 (0.74–2.26)	.371	2.49 (1.25–4.93)	.009
Source				
GI vs. GU	0.45 (0.24–0.87)	.017	0.29 (0.14–0.62)	.001
Line-related vs. GU	0.19 (0.08–0.49)	< .001	0.12 (0.04–0.32)	< .001
SSTI vs. GU	0.68 (0.04–11.3)	.784	–	–
Unclear vs. GU	0.37 (0.12–1.12)	.08	–	–

OR, odds ratio; aOR, adjusted odds ratio; CI, confidence interval.

## Discussion

In this multicenter, retrospective cohort study of adults with uncomplicated enterococcal bacteremia, patients who received early PO antibiotics had similar clinical outcomes compared with those treated with IV therapy alone. No significant differences were observed in clinical cure, treatment failure, infection-related readmissions, or 90-day mortality between the two groups. These findings support the growing body of literature suggesting that early PO transition is a safe and effective treatment strategy for select patients with uncomplicated enterococcal bloodstream infections.

Baseline characteristics were largely comparable between the two groups, although patients with GU infection sources and those with *E. faecium* infections were more frequently transitioned to oral therapy, whereas patients with GI or line-related infections, as well as those with *E. faecalis* bacteremia, were more often maintained on IV therapy. Patients were less likely to transition to early PO antibiotics if they had more severe illness, ID consult, or a non-GU source of bacteremia. The association between ID consultation and lower odds of PO transition should be interpreted cautiously, as it likely reflects confounding by indication rather than direct effect. Patients receiving ID consult may have greater clinical complexity, higher perceived risk, uncertain source control, or other factors favoring continued IV therapy. We included patients with *E. faecium* bacteremia which differs from some similar studies, such as Al Mansi et al. and Loudermilk et al., which focused exclusively on *E. faecalis*.^4,14^ Inclusion of all species strengthens the generalizability of our findings and reflects real-world clinical practice, where *E. faecium* often poses management challenges due to limited IV options and variable susceptibility profiles.

Emerging literature has begun to explore the feasibility of oral antibiotics in immunocompromised hosts. Nussbaum et al reported that early oral transition in solid organ transplant recipients with uncomplicated gram-negative bacteremia resulted in similar outcomes and treatment duration compared with continued IV therapy and emphasized the importance of using highly bioavailable agents.^15^ Our cohort had a relatively high proportion of patients with immunocompromising conditions (41.1%), and our results show that oral antibiotics were not universally avoided in these higher-risk populations. Of note, no HSCT recipients received early PO antibiotics. To our knowledge, this is the largest cohort of immunocompromised conditions on this topic, although the mix of included conditions was quite diverse and consisted mostly of solid tumor malignancies with chemotherapy in the past 90 days. Clinical outcomes were similar in both the IV and early PO transition groups, and there was not a statistically significant association between immunocompromising status and early transition to PO therapy in our multivariable analysis (aOR 0.85, 95% CI 0.45–1.58, *P* = .600). Although our study was not powered to evaluate outcomes within this subgroup, our findings offer preliminary support that oral therapy may be safe in carefully selected immunocompromised patients who are clinical stability, have adequate source control, and show no concerns for malabsorption.

Treatment failure was uncommon and did not differ significantly between groups. This finding is broadly consistent with prior evaluations of oral therapy for enterococcal bacteremia, although direct comparison is limited by differences in study populations and outcome definitions. Al Mansi et al. defined treatment failure as all-cause mortality or *E. faecalis* bloodstream recurrence within 90 days, reporting rates of 14.5% of patients in the PO group and 24% in the IV group (*P* = .19). Loudermilk et al. used a composite definition of 30-day mortality, microbiologic failure, or clinical failure, with treatment failure rates of 14.5% in the PO group and 21.8% in the IV group (OR 0.53; 95% CI 0.23–1.25). In contrast, our definition incorporated provider-assessed clinical non-improvement, infection recurrence, and/or clinical worsening prompting a change in therapy, with clinical cure absent in 26.1% of IV therapy patients and 17.8% of those receiving early PO antibiotics. Although the heterogeneity in definitions complicates direct comparisons, the overall trends across studies suggest that PO therapy achieves outcomes comparable to IV therapy. Safety outcomes were also similar between studies, with a low overall incidence of antibiotic associated adverse effects.

Our findings suggest that clinicians typically prescribe approximately two weeks of therapy regardless of antibiotic route. In the early PO transition group, patients received a median of 5 days of IV therapy before oral transition, consistent with prior studies.^4,14^ While collectively these findings reflect current clinical practice for uncomplicated enterococcal bacteremia, there is growing interest in shortening total duration for uncomplicated infections more broadly. Zimmermann et al. found no difference in outcomes among patients receiving shorter (4–10 d) versus longer (11–18 d) courses for uncomplicated enterococcal bacteremia.^16^ Similarly, the BALANCE trial, where enterococcal bacteremia accounted for 6.9% of the cohort, demonstrated that 7 days of antibiotic therapy was noninferior to 14 days for non-*Staphylococcus aureus*, non-invasive bloodstream infections, although severely immunocompromised patients were excluded.^17^ The ongoing, randomized, prospective INTENSE trial will provide prospective data comparing 7 versus 14 days specifically for uncomplicated enterococcal bacteremia.^5^ If 7 days of therapy proves sufficient, the next question may be whether even earlier oral transition preserves outcomes.

Linezolid was the most frequently prescribed oral agent, followed by amoxicillin-based regimens. The predominant use of linezolid is consistent with its excellent oral bioavailability, established efficacy in enterococcal infections, and ease of dosing. Notably, *E. faecium* infections were more frequently managed with oral linezolid, likely reflecting its favorable susceptibility and concerns regarding daptomycin target attainment in isolates with higher MICs.^18^ Amoxicillin was utilized when susceptibility allowed, typically at 1 gram every 8 hours, supporting oral beta-lactams as a viable option in select cases and highlights using higher doses to improve oral bioavailability. The diversity of oral agents prescribed ultimately underscores real-world flexibility in PO transition approaches, although comparative effectiveness between regimens has not been systematically evaluated.

Lastly, consistent with prior evaluations of early PO transition for uncomplicated gram-positive bloodstream infections,^4,7,8,14^ early PO transition was associated with shorter HLOS compared with IV therapy alone. This reduction has important implications for improving patient quality of life, reducing the risk of hospital-acquired infection and associated morbidity, lowering overall total healthcare costs, and optimizing allocation of healthcare resources.^19,20^ Importantly, these benefits were not associated with increased adverse effects, infection-related readmissions, or IV line complications.

### Limitations and future directions

This study has several important limitations. As a retrospective analysis, it is subject to unmeasured confounding, documentation bias, and selection bias, particularly regarding which patients were selected for oral therapy. Diagnostic evaluation was determined by clinician documentation and practice patterns, and incomplete or unrecognized evaluations may not have been fully captured. Missing data are possible, particularly for recurrence or readmissions, since hospitalizations at outside institutions may not have been connected to the patient’s profile in our EMR. Patients who died before completing therapy were excluded, which may have skewed outcomes toward those with less severe illness; however, this approach is consistent with prior retrospective studies as these patients likely would not have been suitable candidates for early PO transition.^7^ Although we evaluated factors associated with early PO transition, treatment failure was uncommon, and the study was not powered to identify predictors of unsuccessful oral transition. Generalizability to profoundly immunosuppressed populations is also limited because immunocompromising conditions were heterogeneous, and HSCT recipients and patients with hematologic malignancies were underrepresented among those transitioned to oral therapy; these findings should not be generalized to such populations without further study. Lastly, postdischarge adherence could not be fully assessed, and antibiotic selection and dosing varied across sites, potentially confounding efficacy comparisons between the two groups.

## Conclusion

Early PO transition for uncomplicated enterococcal bacteremia was associated with similar clinical outcomes and a shorter HLOS compared with IV therapy alone. Clinical stability, clearance of bacteremia within 48 hours, adequate source control, absence of persistent or endovascular infection, availability of a highly bioavailable oral option, and the ability to tolerate and adhere to oral therapy are all important factors to consider. Although these criteria are based on expert opinion rather than prospective validation, they may provide a practical framework for patient selection. Overall, our results suggest that early PO transition may be safe and effective in select patients with uncomplicated enterococcal bacteremia and underscore the need for prospective studies to confirm optimal treatment duration and refine selection criteria for early oral therapy.

## Table 4 Long description

A table comparing univariate and multivariate model results for predictors of early PO transition. The table has 12 rows and 5 columns. The columns are labeled Variable, Univariate model OR with 95% CI, Univariate model P-value, Multivariate model aOR with 95% CI, and Multivariate model P-value. The rows are labeled Male sex, Age (per year), BMI (per unit), Pitt bacteremia score (per point), Immunocompromising condition, Renal dysfunction (Mild vs. none, Moderate vs. none, Severe vs. none), ID consultation, Pathogen (E. faecium vs. E. faecalis), and Source (GI vs. GU, Line-related vs. GU, SSTI vs. GU, Unclear vs. GU). The table lists the OR with 95% CI and P-value for the univariate model, and the aOR with 95% CI and P-value for the multivariate model for each variable.

Navigate back to Table 4.
